# Toward Precision Biodiversity Detection: An Edge‐Deployable Framework for Mitigating Data Redundancy

**DOI:** 10.1002/ece3.73927

**Published:** 2026-06-28

**Authors:** Huihui Sun, Yang Liu, Yang Wang

**Affiliations:** ^1^ School of Mechanical and Electrical Engineering Huainan Normal University Huainan China; ^2^ School of Technology Beijing Forestry University Beijing China

**Keywords:** biodiversity, edge computing, lightweight model, object recognition, wildlife detection

## Abstract

Monitoring wildlife in remote areas is vital for biodiversity research, yet infrared‐triggered cameras often produce large numbers of false or empty images due to environmental interference, causing data overload and power waste. To address this, this manuscript proposes WS‐YOLO, a lightweight edge‐optimized wildlife detection model based on improved YOLOv11n, which aims to integrate false‐trigger filtering and accurate animal detection in a single model for field applications. Specifically, the model introduces three key enhancements: it adopts a Wavelet Convolution (WTConv) module to capture multi‐scale and frequency‐aware features, boosting robustness to complex backgrounds and small‐target detection accuracy; meanwhile, a Spatial‐Channel Synergistic Attention (SCSA) mechanism is incorporated to enhance spatial localization and channel‐wise semantic encoding, enabling the model to focus on salient regions of wildlife; additionally, a SlideLoss function is designed to prioritize training on boundary cases, improving the handling of ambiguous detection instances. Experiments on four wildlife datasets demonstrate that WS‐YOLO achieves excellent mAP@0.50 performance: 93.0% on African Wildlife, 98.0% on Amur Tiger, 98.5% on the NTLNP infrared dataset, and 79.4% on PASCAL VOC, confirming its robustness across diverse scenarios. Deployment on a Jetson Nano further validates its feasibility for real‐time, energy‐efficient use in the field. WS‐YOLO enables accurate animal recognition and classification directly at edge devices, significantly reducing communication load and enhancing monitoring efficiency.

## Graphical abstract

This research introduces the WS—YOLO, a lightweight and high—efficiency wildlife detection model that is specifically optimized for edge deployment within camera trap systems. The goal is to enable both false trigger filtering and accurate animal detection within a single model, tailored for low‐power edge deployment. The proposed method contributes to the broader trend toward autonomous, energy‐aware, and intelligent ecological monitoring systems worldwide.

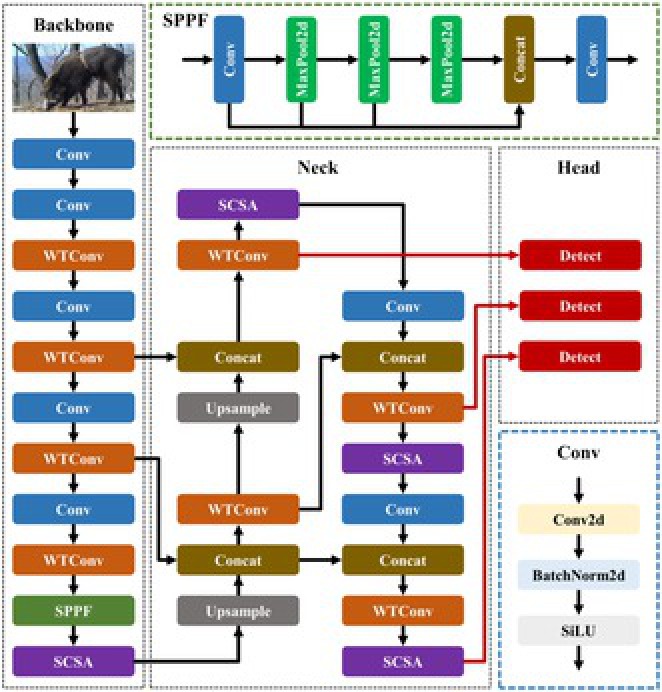

## 1 Introduction

Long‐term wildlife monitoring is a fundamental component of biodiversity conservation and ecological research. Infrared‐triggered camera traps, due to their noninvasive nature, cost‐effectiveness, and ability to operate continuously in all weather conditions, have become one of the most widely adopted tools for documenting species distributions, behavioral patterns, and activity rhythms in the wild (Bruce et al. 2025; Pestell et al. 2025). With recent advancements in wireless communication technologies, some researchers have developed camera systems capable of ad hoc networking and autonomous data transmission in areas lacking public network infrastructure (Wei et al. 2020; Liu et al. 2019). These systems have demonstrated strong adaptability and real‐time performance in various complex environments.

However, one persistent issue in field deployment of camera traps is the high proportion of false triggers and empty frames, where no animals are actually captured. Such false activations in camera traps networks frequently arise from environmental disturbances, including moving vegetation, sudden lighting changes, or thermal interference (Newey et al. 2015; Norouzzadeh et al. 2018). These ineffective images not only consume valuable network bandwidth, but also significantly increase energy usage, reducing device longevity and data transmission efficiency (Zualkernan et al. 2022). For battery‐powered edge devices in particular, the frequent transmission of empty images can severely shorten operational periods and hinder monitoring effectiveness. Therefore, integrating intelligent image filtering and wildlife recognition directly into the camera system—prior to data transmission—presents a promising solution to reduce data redundancy and energy consumption.

The convergence of edge computing and deep learning in recent years has enabled lightweight object detection models to run in real time on low‐power platforms, offering powerful tools for intelligent ecological monitoring (Panigrahi et al. 2023). Among these models, the You Only Look Once (YOLO) series (Redmon et al. 2016; Redmon and Farhadi 2017; Chung et al. 2025; Bochkovskiy et al. 2020; Jocher et al. 2025; Wang et al. 2023) has been widely adopted for its simplicity, speed, and detection accuracy. In ecological applications, Lu et al. (2024) proposed OA‐YOLO, introducing refined objectness scoring and a novel label assignment strategy, significantly improving wildlife detection accuracy. Pestell et al. (2025) highlighted the potential for improving YOLO‐based object detection in wildlife monitoring by employing custom‐built smart camera traps (DeakinCams) and diverse training datasets, particularly for detecting small and ectothermic species. These studies confirm that deep learning models are capable of extracting animal targets from complex natural backgrounds.

From a deployment perspective, edge devices such as the NVIDIA Jetson Nano and Raspberry Pi have been increasingly utilized in field monitoring systems. Vasavi et al. (2025) proposed a transformer‐based detection framework, deployable on Jetson Nano, achieving high‐accuracy (90% F1, 83% mAP) real‐time detection and tracking of whales from satellite imagery. Liu et al. (2025) proposed LFN‐YOLO, a lightweight object detection model optimized for underwater environments, which integrates RepGhost, SPD‐Conv, GFPN, and CLLAHead to enhance small object detection and multi‐scale feature fusion, achieving high accuracy with low computational cost on Jetson AGX Orin. Wu et al. (2025). introduced AnimalRTPose, a high‐speed, one‐stage cross‐species animal pose estimation model that integrates CSPNeXt, CAM, and SPP for efficient multi‐scale feature extraction, achieving real‐time performance on both GPUs and Raspberry Pi 5, making it well‐suited for real‐time animal behavior monitoring. While these pioneering studies laid the groundwork for edge‐based intelligent filtering, challenges remain—particularly in generalizing models across species and maintaining detection accuracy post‐deployment.

To address these limitations, this study proposes a novel wildlife detection model, termed WS‐YOLO, based on improvements to the YOLOv11n framework. The goal is to enable both false trigger filtering and accurate animal detection within a single model, tailored for low‐power edge deployment. Specifically, this study introduces three key enhancements: (1) a Wavelet Convolution (WTConv) module to capture multi‐scale and frequency‐aware representations, improving robustness to complex backgrounds and small object detection; (2) a Spatial and Channel Synergistic Attention (SCSA) mechanism to jointly enhance spatial localization and channel‐wise semantic encoding; and (3) a SlideLoss function that adaptively focuses training on boundary cases, such as animals under partial occlusion or poor lighting conditions.

To comprehensively evaluate the model's performance and generalizability, we conducted experiments on four representative datasets: the African Wildlife (AW) dataset, the Amur Tiger image set, the NTLNP infrared dataset collected in Northeast China, and the public PASCAL VOC benchmark. The results show that WS‐YOLO outperforms other state‐of‐the‐art lightweight detectors in terms of false trigger suppression, detection accuracy, and real‐time inference speed. Moreover, real‐world deployment on the Jetson Nano platform demonstrates the model's energy efficiency, low latency, and robustness in practical edge computing scenarios.

This study presents a viable approach for intelligent upgrades to infrared‐triggered camera systems, significantly reducing unnecessary data transmission and increasing the value density of collected information. The proposed method contributes to the broader trend toward autonomous, energy‐aware, and intelligent ecological monitoring systems worldwide.

## 2 Materials and Methods

### 2.1 Datasets

To validate the effectiveness and generalizability of the proposed WS‐YOLO model, we leveraged four representative wildlife image datasets.

#### 2.1.1 African Wildlife Dataset

The AW Dataset (Ultralytics 2025) is an openly available camera‐trap collection comprising approximately 1500 annotated images, distributed across four African mammal species: buffalo, elephant, rhino, and zebra. Images were captured in natural savanna environments under varying lighting and seasonal conditions, yielding both clear and partially occluded subject instances. The dataset features moderate intra‐class variance and occlusion scenarios, making it suitable for assessing generalizability and small‐object detection capability in wildlife detection tasks.

#### 2.1.2 Amur Tiger Dataset

The Amur Tiger Dataset used in this study is based on the detection subset of the ATRW (Amur Tiger Re‐identification in the Wild) dataset (Ma et al. 2025). It contains annotated images of wild Amur tigers captured by infrared‐triggered camera traps in the dense forests of Northeastern China. The dataset features complex natural scenes with high levels of background clutter, heavy vegetation, and frequent partial occlusion. One of its notable characteristics is the strong class imbalance, with relatively few tiger instances compared to the large number of background and nontarget frames. This presents a realistic and challenging setting for evaluating object detection algorithms, particularly in low‐shot and imbalanced conditions.

#### 2.1.3 Northeast Tiger and Leopard National Park Dataset

The Northeast Tiger and Leopard National Park (NTLNP) dataset (Tan et al. 2022) was constructed from infrared‐triggered camera trap footage collected between 2014 and 2020 in the mixed coniferous–broadleaf forests of Northeast China, marking the first large‐scale dataset of its kind in this region. A total of approximately 25,657 images were extracted from video clips and annotated in PASCAL‐VOC format. Seventeen animal species are represented, including 15 wild mammals (such as Amur tiger, Amur leopard, wild boar, roe deer, sika deer, and Asian black bear) and two domesticated animals, offering a rich variety of both large and small species. The dataset includes daytime and nighttime infrared imagery, presenting significant challenges such as strong background clutter, varied illumination, and class imbalance, with relatively few target animal frames compared to abundant empty or non‐target scenes. This dataset provides a realistic and demanding benchmark to evaluate object detection models for wildlife recognition under authentic field conditions.

#### 2.1.4 Visual Object Classes Dataset

The Visual Object Classes (VOC) dataset (Everingham et al. 2010) is a widely used benchmark in computer vision, especially for object detection and image segmentation. In this study, we adopted both the VOC2007 and VOC2012 versions, which include 9963 and 11,540 annotated images, respectively, covering 20 common object categories such as person, dog, car, and bicycle. Although not focused on wildlife, the VOC dataset serves as a valuable benchmark to evaluate the generalization capability of detection models in diverse visual contexts.

### 2.2 Model Architecture: WS‐YOLO

**FIGURE 1:**
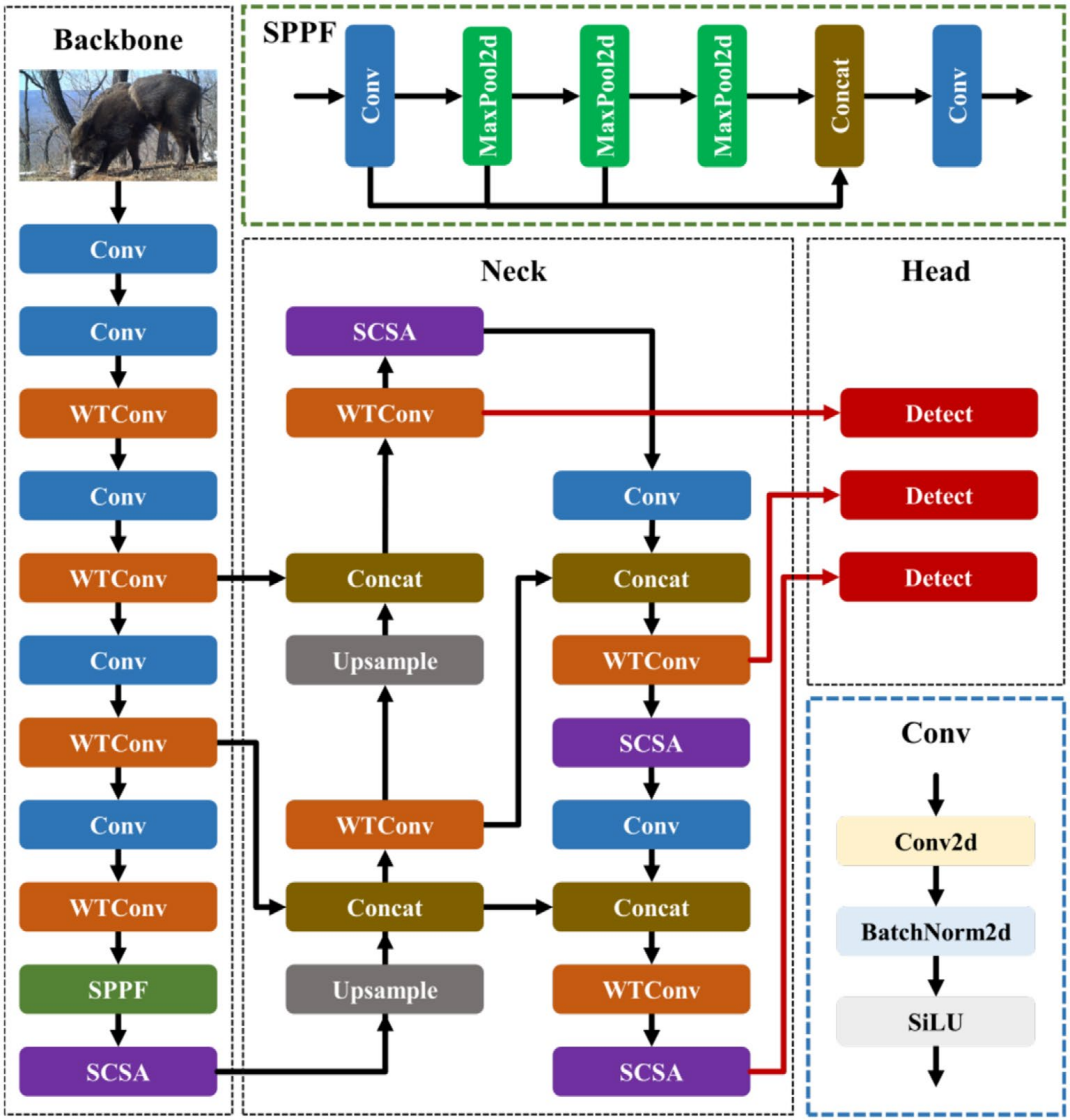
The architecture of the proposed WS‐YOLO model, illustrating the integration of WTConv, SCSA, and SlideLoss.

WS‐YOLO is a lightweight object detection model designed to balance detection performance with computational efficiency for edge device deployment. It builds upon the YOLOv11n baseline with three key enhancements: WTConv, SCSA, and a customized adaptive loss function named SlideLoss. The structure diagram of WS‐YOLO is shown in Figure 1.

#### 2.2.1 Wavelet Convolution

**FIGURE 2:**
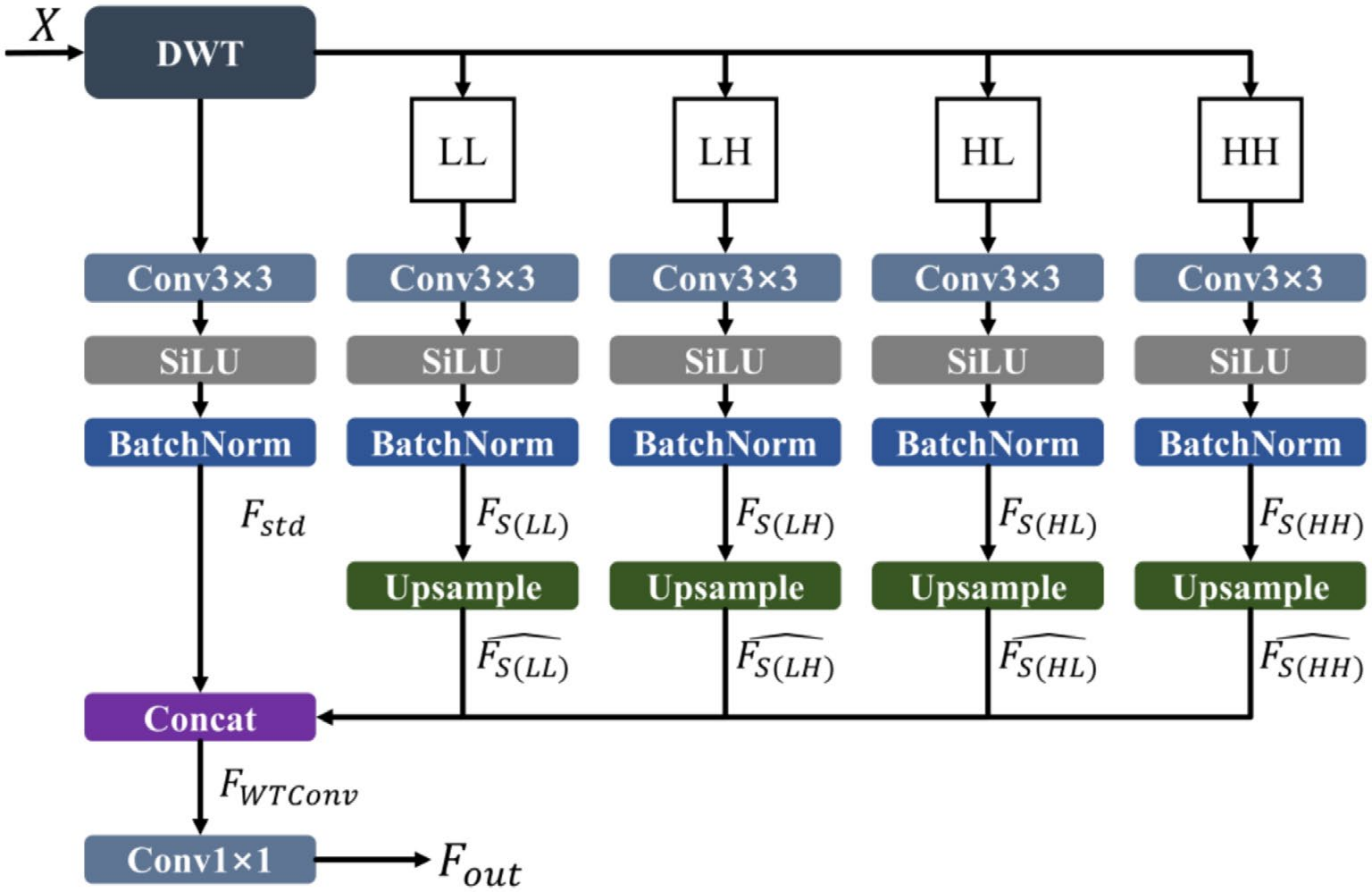
The architecture of WTConv. It includes low‐ and high frequency decomposition, followed by convolution and upsampling for enhanced feature fusion.

In natural wildlife monitoring scenarios, animal targets often appear in low contrast, small scales, or partially occluded regions. Traditional convolutional neural networks (CNNs), operating solely in the spatial domain, may struggle to extract sufficient discriminative features under such conditions. To address this, we introduce WTConv (Finder et al. 2024) into the backbone and neck of WS‐YOLO, enabling the joint extraction of spatial and frequency‐domain information through discrete wavelet decomposition. Figure 2 shows the processing flow of WTConv.

Let the input feature map be denoted as $X\in {\mathbb{R}}^{C\times H\times W}$. A two‐dimensional Discrete Wavelet Transform (DWT) is applied channel‐wise to decompose the feature map into four subbands:

(1) $$\mathrm{DWT}\;\left(X\right)=\left\{\mathrm{LL},\mathrm{LH},\mathrm{HL},\mathrm{HH}\right\}$$

where LL denotes the low frequency approximation (global features), while LH, HL, and HH denote the horizontal, vertical, and diagonal high frequency components, respectively. All subbands are of shape ${\mathbb{R}}^{C\times \frac{H}{2}\times \frac{W}{2}}$.

Each subband is processed independently using a standard convolutional block (convolution, batch normalization, and activation), denoted as:

(2) $$F_{S}=\mathrm{BN}\left(\sigma \left({\text{Conv}}_{3\times 3}\left(S\right)\right)\right),S\in \left\{\mathrm{LL},\mathrm{LH},\mathrm{HL},\mathrm{HH}\right\}$$

where σ is an activation function, e.g., SiLU or ReLU.

To align with the original spatial dimensions, each feature map is upsampled back to H×W using nearest‐neighbor interpolation:

(3) $$\hat{F_{S}}=\text{Upsample}\left(F_{S}\right),\hat{F_{S}}\in {\mathbb{R}}^{C^{\prime }\times H\times W}$$

Simultaneously, the original input X undergoes a spatial‐domain convolution:

(4) $$F_{\mathrm{std}}=\mathrm{BN}\left(\sigma \left({\text{Conv}}_{3\times 3}\left(X\right)\right)\right)$$

The WTConv output is obtained by concatenating all five processed branches:

(5) $$F_{\text{WTConv}}=\text{Concat}\left[F_{\mathrm{std}},{\hat{F}}_{\mathrm{LL}},{\hat{F}}_{\mathrm{LH}},{\hat{F}}_{\mathrm{HL}},{\hat{F}}_{\mathrm{HH}}\right]$$

To compress the resulting feature volume and enable efficient downstream computation, a 1×1 convolution is applied:

(6) $$F_{\text{out}}={\text{Conv}}_{1\times 1}\left(F_{\text{WTConv}}\right)$$

This design allows the model to benefit from complementary representations: the global context from low frequency signals, and the textural and edge cues from high frequency components. In practice, this significantly enhances the feature richness in shallow and mid‐level layers—where small‐scale targets and fine‐grained environmental textures are most critical.

In the WS‐YOLO architecture, WTConv replaces two conventional convolutional layers in the backbone, specifically in early stages where feature resolution is highest. This strategic integration enables effective preservation and enhancement of object boundaries, contours, and frequency‐based discriminators, laying a solid foundation for downstream attention modeling and detection refinement.

#### 2.2.2 Spatial and Channel Synergistic Attention

**FIGURE 3:**
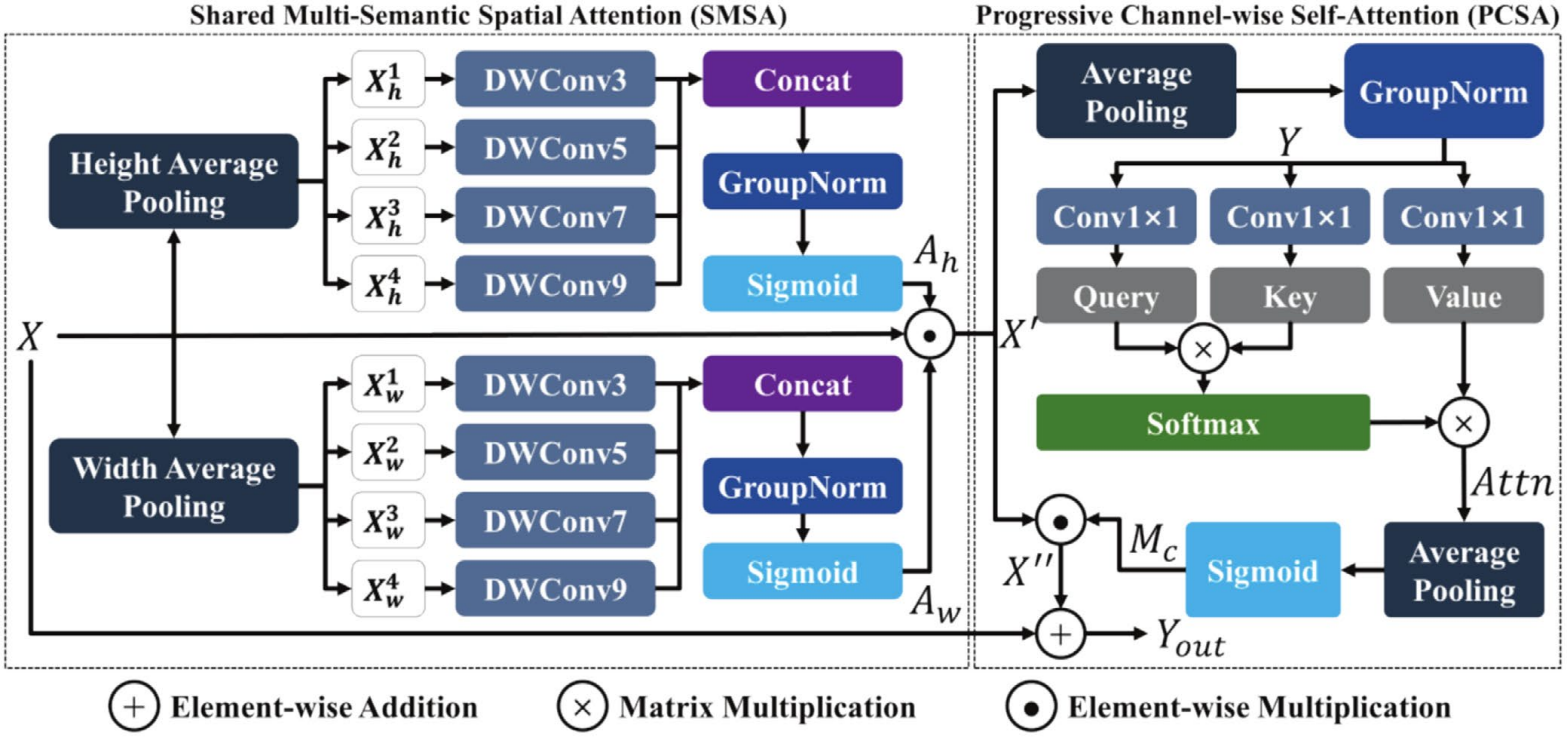
The architecture of the Spatial and Channel Synergistic Attention (SCSA) module, consisting of spatial attention (left) and channel attention (right) branches for enhanced feature representation.

To enhance feature representation under challenging wildlife detection scenarios (e.g., occlusion, small scale, low contrast), we integrate a SCSA (Si et al. 2025) module into our detection backbone. Unlike conventional attention mechanisms that treat spatial and channel dimensions independently, SCSA performs sequential spatial and channel attention modeling in a mutually reinforcing manner. The structure diagram of SCSA is shown in Figure 3.

Let the input feature map be denoted as:

(7) $$X\in {\mathbb{R}}^{B\times C\times H\times W}$$

where B is the batch size, C is the number of channels, and H×W is the spatial resolution.

The spatial attention module captures long‐range dependencies along both height and width dimensions. First, average pooling is applied across width and height to obtain spatial context descriptors:

(8) $$X_{h}=\frac{1}{W}\sum_{i=1}^{W}X_{:,:,:,i},i\in {\mathbb{R}}^{B\times C\times H},X_{w}=\frac{1}{H}\sum_{j=1}^{H}X_{:,:,j,:},j\in {\mathbb{R}}^{B\times C\times W}$$

Each descriptor is divided into four groups along the channel dimension:

(9) $$X_{h}=\left[X_{h}^{1},X_{h}^{2},X_{h}^{3},X_{h}^{4}\right],X_{w}=\left[X_{w}^{1},X_{w}^{2},X_{w}^{3},X_{w}^{4}\right]$$

Each group is processed using different depth‐wise 1D convolutions with varying kernel sizes $k\in \left\{3,5,7,9\right\}$, followed by group normalization and a sigmoid gate:

(10) $$A_{h}=\sigma \;\left(\mathrm{GN}\left[{\text{DWConv}}_{3\times 3}\left(X_{h}^{1}\right)|\left|{\text{DWConv}}_{5\times 5}\left(X_{h}^{2}\right)\right||{\text{DWConv}}_{7\times 7}\left(X_{h}^{3}\right)\|{\text{DWConv}}_{9\times 9}\left(X_{h}^{4}\right)\right]\right)$$

(11) $$A_{w}=\sigma \;\left(\mathrm{GN}\left[{\text{DWConv}}_{3\times 3}\left(X_{w}^{1}\right)|\left|{\text{DWConv}}_{5\times 5}\left(X_{w}^{2}\right)\right||{\text{DWConv}}_{7\times 7}\left(X_{w}^{3}\right)\|{\text{DWConv}}_{9\times 9}\left(X_{w}^{4}\right)\right]\right)$$

The attention maps $A_{h}\in {\mathbb{R}}^{B\times C\times H\times 1}$ and $A_{w}\in {\mathbb{R}}^{B\times C\times 1\times W}$ are reshaped and broadcasted to modulate the original feature map:

(12) $$X^{\prime }=X⊙A_{h}⊙A_{w}$$

To capture global interchannel dependencies, a downsampled version of $X^{\prime }$ is first obtained using spatial pooling:

(13) $$Y=\text{Down}\left(X^{\prime }\right),Y\in {\mathbb{R}}^{B\times C\times h^{\prime }\times w^{\prime }}$$

The downsampled features are normalized and used to compute self‐attention in the channel dimension. Queries, keys, and values are generated using grouped 1×1 convolutions:

(14) $$Q={\text{Conv}}_{1\times 1}\left(Y\right),K={\text{Conv}}_{1\times 1}\left(Y\right),V={\text{Conv}}_{1\times 1}\left(Y\right)$$

After reshaping for multi‐head attention, scaled dot product attention is computed:

(15) $$\text{Attn}=\text{Softmax}\left(\frac{QK^{T}}{\sqrt{C}}\right)V$$

The attended features are pooled across spatial dimensions to yield a channel attention map:

(16) $$M_{C}=\sigma \left(\frac{1}{h^{\prime }w^{\prime }}\sum_{i=1}^{h^{\prime }}\sum_{j=1}^{w^{\prime }}{\text{Attn}}_{:,:,i,j}\right)\in {\mathbb{R}}^{B\times C\times 1\times 1}$$

Finally, the output of the SCSA module is obtained via element‐wise multiplication and residual connection:

(17) $$X^{\prime \prime }=X^{\prime }⊙M_{C},Y_{\text{out}}=X^{\prime \prime }+X$$

The SCSA module is integrated into the end of the backbone and neck of the network, particularly in the feature fusion stages. By jointly modeling spatial and channel attention in a cascaded manner, SCSA enables the network to better focus on discriminative object regions while suppressing irrelevant background noise. This is especially beneficial in complex environments with occluded or camouflaged wildlife targets.

#### 2.2.3 Adaptive Sliding Gradient Loss Function

In wildlife detection tasks, the imbalance between foreground animal targets and large background regions—especially in infrared camera‐trap images—presents a persistent challenge. Additionally, many target instances exhibit partial occlusion, motion blur, or low contrast, resulting in ambiguous boundaries and making them hard to learn using conventional loss functions. To address these challenges, we propose Adaptive Sliding Gradient Loss Function (SlideLoss) (Yu et al. 2024), a novel adaptive sliding gradient loss function designed to enhance the network's ability to focus on ambiguous samples and difficult regions while maintaining stable convergence.

Standard loss functions such as Binary Cross Entropy (BCE) or Focal Loss treat each sample independently and assign fixed weights, which is suboptimal in longtail or partially observable conditions. Focal Loss, though designed to mitigate class imbalance, still applies uniform treatment to all hard samples, regardless of their spatial or semantic context. Inspired by margin‐based learning and dynamic modulation principles, SlideLoss introduces a sliding margin mechanism, allowing the model to dynamically focus on uncertain samples with flexible sensitivity.

Let $p\in \left[0,1\right]$ be the predicted objectness score for a sample, and $y\in \left\{0,1\right\}$ be the ground truth label. The base loss is derived from standard BCE loss:

(18) $$L_{\mathrm{BCE}}\left(p,y\right)=-\left[y\log\;\left(p\right)+\left(1-y\right)\;\log\;\left(1-p\right)\right]$$

We introduce a sliding margin δ and a modulation function $\emptyset \left(p,y,\delta \right)$ to scale the gradient based on sample uncertainty:

(19) $$\emptyset \left(p,y,\delta \right)=\left\{\begin{array}{c} {\left(1-p-\delta \right)}^{\gamma },y=1,p<1-\delta \\ {\left(p-\delta \right)}^{\gamma },y=0,p>\delta \\ 1,\text{otherwise} \end{array}\right.$$

where γ>0 is a focusing parameter (similar to Focal Loss), $\delta \in \left(0,0.5\right)$ is the adaptive sliding margin.

The complete SlideLoss is then expressed as:

(20) $$L_{\text{Slide}}\left(p,y\right)=\emptyset \left(p,y,\delta \right)\cdot L_{\mathrm{BCE}}\left(p,y\right)$$

This formulation allows the loss to emphasize:

- False negatives: when y=1 but p is far below 1;
- False positives: when y=0 but p is still too high;
- And to down‐weight easy examples when predictions lie within an acceptable confidence band.

Unlike fixed‐margin methods, we dynamically adjust δ during training based on batch‐level uncertainty statistics. Specifically, we compute:

(21) $$\delta =\alpha \cdot \text{StdDev}\left(p\right),\alpha \in \left[0.1,0.5\right]$$

where $\text{StdDev}\left(p\right)$ denotes the standard deviation of the predicted confidence scores across all positive and negative anchors within a mini‐batch. This adaptive mechanism allows SlideLoss to apply a larger margin during the early stages of training—when prediction variance is high—and gradually reduce it as the model converges, thereby focusing on finer error correction.

In WS‐YOLO, SlideLoss is applied to both the objectness prediction and classification branches. For bounding box regression, we retain CIoU Loss for its spatial interpretability. SlideLoss serves as a plug‐in module that replaces standard BCE/Focal Loss in the classification and confidence outputs without architectural modification. Its ability to adaptively emphasize hard examples significantly improves the network's robustness in detecting partially visible, small, or camouflaged animals under complex environmental conditions.

### 2.3 Experimental Setup

All training and baseline inference experiments were conducted on an Ubuntu‐based server environment utilizing the PyTorch deep learning framework to ensure consistent performance evaluation and model convergence. To assess the real‐world applicability of the proposed model in resource‐constrained scenarios, we further deployed it on an NVIDIA Jetson Nano device. This allowed us to investigate both the inference speed and the impact of input resolution on runtime efficiency under actual edge computing conditions. Comprehensive details regarding the hardware and software environments for both the server and edge platforms are summarized in Table 1.

**TABLE 1:** Configuration of Ubuntu server and NVIDIA Jetson Nano.

Environments	Ubuntu server configuration	NVIDIA Jetson Nano configuration
System	Ubuntu 20.04.1 LTS	Ubuntu 18.04.6 LTS
CPU	Intel(R) Xeon(R) Silver 4310 CPU@2.10GHz	ARM Cortex‐A57 MPCore
GPU	NVIDIA GeForce RTX 3090	NVIDIA Tegra X1 128‐core
RAM	512GB	4GB
Framework	PyTorch 2.1.0	PyTorch 1.11.0
CUDA	12.1	10.2
Compiler	Python 3.9.20	Python 3.8.0

Model training was conducted using input images resized to 640 × 640 pixels. A batch size of 16 was employed, with the optimization process carried out using the Adam algorithm and an initial learning rate set to 0.01. The training procedure was executed for a total of 200 epochs to ensure stable convergence.

### 2.4 Evaluation Metrics

To comprehensively assess the performance of the proposed detection model, a set of evaluation indicators was employed, including precision (*P*), recall (*R*), mean average precision (mAP), frames per second (FPS), floating‐point operations (FLOPs), and model parameter size (Params). These metrics jointly reflect detection accuracy, computational efficiency, and deployment feasibility on resource‐constrained edge devices.

*P* quantifies the proportion of correctly predicted positive instances among all predicted positives, while *R* measures the proportion of correctly identified positives among all actual positives. Formally, they are defined as:

(22) $$P=\frac{\mathrm{TP}}{\mathrm{TP}+\mathrm{FP}},R=\frac{\mathrm{TP}}{\mathrm{TP}+\mathrm{FN}}$$

where TP is the number of true positive predictions, FP is false positives, and FN is false negatives.

The Intersection over Union (IoU) is used to evaluate how closely a predicted bounding box aligns with the corresponding ground truth. It is defined as:

(23) $$\mathrm{IoU}=\frac{\text{Area of Overlap}}{\text{Area of Union}}$$

Based on the precision–recall relationship, Average Precision (AP) is computed for each class by integrating the precision over the recall curve. The mAP is then derived by averaging AP across all target categories:

(24) $$\mathrm{AP}=\int_{0}^{1}P\left(R\right)\mathrm{dR},\mathrm{mAP}=\frac{1}{C}\sum_{i=1}^{C}{\mathrm{AP}}_{i}$$

where C denotes the total number of classes. In this study, we adopt an IoU threshold of 0.50 for evaluation, and report mAP@0.5 as a key metric.

To assess real‐time processing capacity, FPS is calculated as the number of images processed per second:

(25) $$\mathrm{FPS}=\frac{\text{Number of Frames}}{\text{Inference Time}}$$

This metric is particularly important for edge computing scenarios where low‐latency response is required.

FLOPs represent the total number of arithmetic operations required during one forward pass of the network and serve as a proxy for computational complexity. Params refer to the total count of trainable parameters, reflecting the model's memory footprint. In edge device deployments, models with lower FLOPs and Params are favored due to limited computational and power budgets.

## 3 Results

### 3.1 Ablation Experiment

To evaluate the contribution of each proposed component, a set of ablation experiments was carried out using YOLOv11n as the baseline model. The detection performances on the test set of the ablation experiments are shown in Tables 2 and 3. The modules—WTConv, SCSA, and SlideLoss (SL)—were progressively integrated into the baseline. In the experimental results tables, a “√” symbol denotes the inclusion of a specific module. The effect of each individual enhancement on model performance was systematically examined to validate its effectiveness.

**TABLE 2:** The mAP evaluation metric results of the ablation experiments conducted on four datasets, as well as the comparisons of the number of parameters and computational speed.

WTConv	SCSA	SlideLoss	mAP@0.5 AW	mAP@0.5 ATRW	mAP@0.5 NTLNP	mAP@0.5 VOC	Params (M)	FLOPs (G)	FPS
			0.910	0.969	0.977	0.769	2.58	6.3	110.5
√			0.914	0.979	0.981	0.789	2.73	7.1	124.8
	√		0.910	0.975	0.981	0.770	**2.54**	**6.2**	98.4
		√	0.914	0.975	0.978	0.769	2.58	6.3	111.2
√	√		0.922	0.977	0.982	0.783	2.69	7.1	122.1
	√	√	0.928	0.971	0.979	0.792	**2.54**	**6.2**	97.1
√		√	0.914	0.973	0.982	0.773	2.73	7.1	**125.9**
√	√	√	**0.930**	**0.980**	**0.985**	**0.794**	**2.69**	**7.1**	**121.8**

*Note:* The bolded result is the best.

Abbreviations: ATRW, Amur Tiger Dataset; AW, African Wildlife Dataset; NTLNP, Northeast Tiger and Leopard National Park Dataset; VOC, Visual Object Classes Dataset. The same as below.

**TABLE 3:** The results of the P and R evaluation metric results for the ablation experiments conducted on 4 datasets.

WTConv	SCSA	SlideLoss	AW		ATRW		NTLNP		VOC	
			*P*	*R*	*P*	*R*	*P*	*R*	*P*	*R*
			0.902	0.821	0.948	0.941	0.961	0.951	0.768	0.704
√			0.910	0.835	0.965	0.943	0.963	0.955	0.790	0.708
	√		0.910	0.824	0.964	0.945	0.966	0.955	0.772	0.697
		√	0.918	0.841	0.957	0.952	0.962	0.951	0.760	0.705
√	√		**0.922**	0.845	**0.972**	0.947	0.967	0.956	0.794	0.715
	√	√	0.919	0.866	0.963	0.956	0.961	0.956	0.793	0.699
√		√	0.920	0.843	0.968	0.949	0.966	0.961	0.764	0.710
√	√	√	**0.922**	**0.869**	**0.972**	**0.958**	**0.971**	**0.962**	**0.797**	**0.722**

*Note:* The bolded result is the best.

In terms of detection accuracy measured by mAP@0.5, the baseline YOLOv11n model achieved 0.910, 0.969, 0.977, and 0.769 on AW, ATRW, NTLNP and VOC, respectively. Introducing WTConv alone improved mAP@0.5 on the AW and VOC datasets (0.914 and 0.789, respectively), indicating enhanced multifrequency feature extraction, while maintaining a relatively low parameter count (2.73 M) and high inference speed (124.8 FPS). SCSA also brought slight improvements on ATRW and NTLNP but was less effective on VOC, suggesting that spatial‐channel attention benefits more structured or high‐quality data. SlideLoss, when used independently, led to a consistent rise in mAP@0.5 and maintained lightweight characteristics, confirming its effectiveness in addressing ambiguous or low‐quality samples.

Although the integration of the SCSA module led to a slight reduction in both parameter count and FLOPs, a decrease in inference speed (measured in FPS) was observed. This apparent discrepancy arises from the intrinsic computational characteristics of the SCSA mechanism. Specifically, SCSA combines spatial and channel attention, involving operations such as global pooling, per‐channel scaling, tensor reweighting, and multiple nonlinear activations. While these operations are relatively lightweight in terms of arithmetic complexity, they exhibit limited parallelism and sequential dependencies that hinder efficient hardware utilization. Moreover, frequent reshaping and dimensional transformations of feature maps increase memory access overhead and data movement latency. As a result, despite lower theoretical computational cost, the practical execution time is negatively affected, leading to a slight reduction in overall inference speed.

The combination of WTConv and SCSA further improved detection performance, particularly on AW (0.922) and VOC (0.783), highlighting their complementary strengths in enhancing spatial details and refining attention mechanisms. Similarly, SCSA combined with SlideLoss reached 0.928 mAP on AW and 0.792 on VOC, with reduced model size (2.54 M) and computation cost (6.2G FLOPs), showing its suitability for edge environments. Interestingly, WTConv paired with SlideLoss achieved the highest inference speed (125.9 FPS) while still maintaining competitive accuracy, which is advantageous in real‐time deployment scenarios.

The final WS‐YOLO model, integrating all three components, achieved the best overall performance, with mAP@0.5 scores of 0.930, 0.980, 0.985, and 0.794 across AW, ATRW, NTLNP, and VOC, respectively. The model maintains a compact structure (2.69 M parameters), moderate computational complexity (7.1G FLOPs), and real‐time inference speed (121.8 FPS), confirming its balance of accuracy and efficiency.

In addition to mAP, we also examined precision and recall. WTConv alone improved both metrics (AW: *P* = 0.910, *R* = 0.835), while SCSA contributed to higher recall on ATRW (*R* = 0.945) and SlideLoss improved recall on VOC (*R* = 0.705). The combined modules further enhanced these metrics, with the full WS‐YOLO model achieving the highest precision and recall in most cases (e.g., AW: *P* = 0.922, *R* = 0.869; ATRW: *P* = 0.972, *R* = 0.958; NTLNP: *P* = 0.971, *R* = 0.962; VOC: *P* = 0.797, *R* = 0.722). These results confirm that the proposed modules jointly contribute to more accurate and robust detection, particularly in challenging wildlife monitoring scenarios with limited power and bandwidth.

**FIGURE 4:**
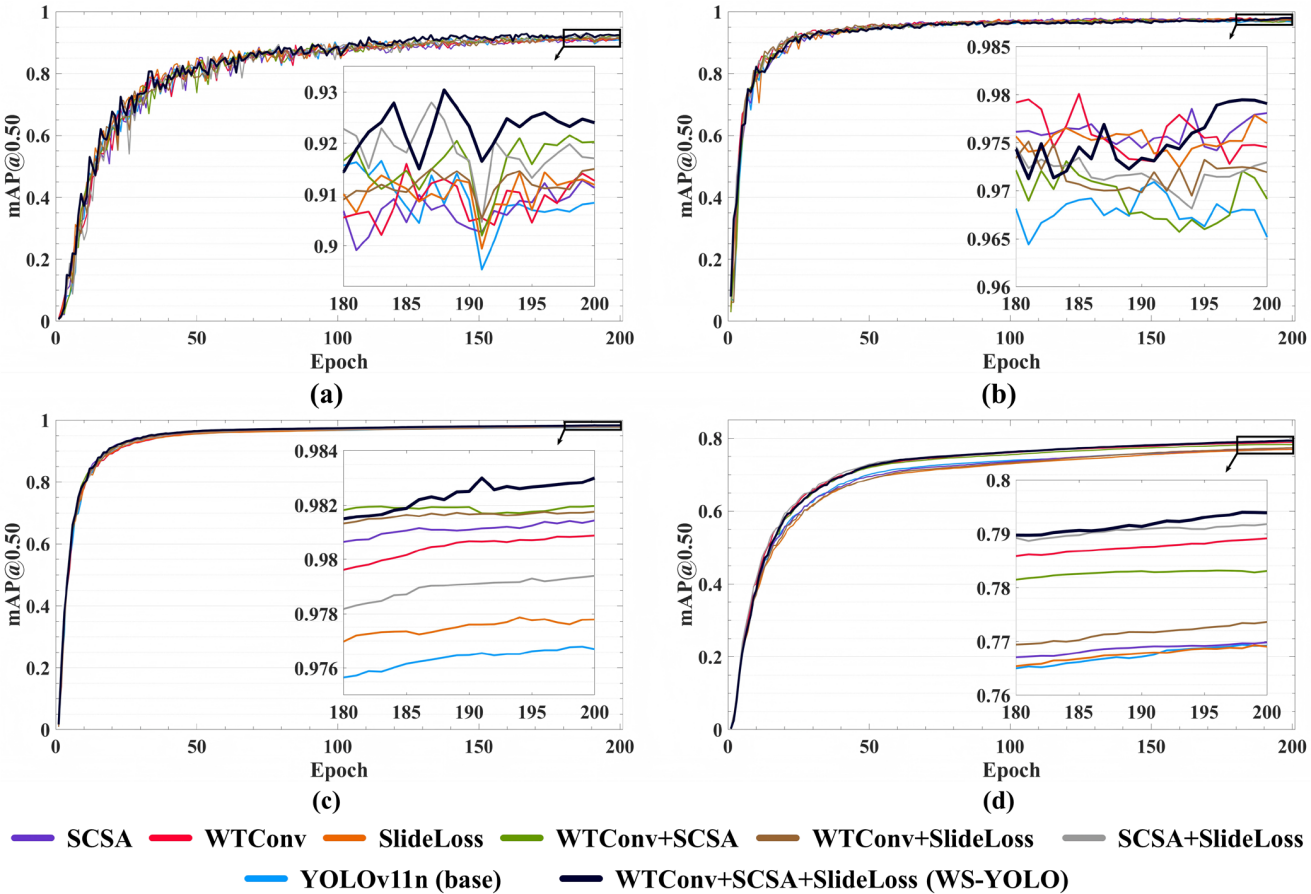
The curve graph of mAP@0.5 changes during the training process: (a) On African Wildlife Dataset; (b) On Amur Tiger Dataset; (c) On Northeast Tiger and Leopard National Park Dataset; (d) On Visual Object Classes Dataset.

Figure 4 illustrates the mAP@0.50 progression across training epochs for different model variants on four benchmark datasets, highlighting the impact of each module on convergence behavior and final detection performance. Throughout the training process, all variants of the model exhibit a similar learning trajectory, with a steep increase in mAP@0.50 during the initial epochs, followed by a slower, more stable convergence phase as training progresses. Notably, the WS‐YOLO model incorporating all three enhancements—WTConv, SCSA, and SlideLoss—consistently outperforms other variants in terms of final detection accuracy across all four datasets. Its mAP@0.50 curve not only reaches a higher plateau but also demonstrates less fluctuation in the later epochs, indicating improved convergence stability and robustness.

Variants with single or partial enhancements show intermediate performance, with marginal improvements over the baseline, but their training curves often display greater variance or slower gains during late‐stage optimization. The improvements brought by WTConv and SCSA are especially evident in the mid‐to‐late training stages, while the introduction of SlideLoss contributes to smoother convergence and better handling of difficult samples.

These results suggest that the full WS‐YOLO architecture leads to more reliable and generalized learning behavior, particularly in complex and variable wildlife detection scenarios.

### 3.2 Model Comparison Experiment

To validate the effectiveness of WS‐YOLO, we compared its performance with several representative object detection models, including the two‐stage Faster R‐CNN (Ren et al. 2017), the one‐stage SSD (Liu et al. 2016), multiple versions from the YOLO series (Chung et al. 2025; Jocher et al. 2025; Thu‐Mig 2025; Wang et al. 2024; Meituan 2025) (including YOLOX (Megvii‐Basedetection 2025)), and the Transformer‐based Swin Transformer (Liu et al. 2021). To ensure a fair and meaningful comparison within the YOLO family, we selected variants whose model sizes and computational complexity are comparable to the YOLOv11n baseline, upon which WS‐YOLO is built.

#### 3.2.1 Performance Comparison

The results across four datasets (AW, ATRW, NTLNP, and VOC) are summarized in Tables 4 and 5. In terms of mAP@0.5, WS‐YOLO achieved top‐tier performance, reaching 0.930, 0.980, 0.985, and 0.794 on AW, ATRW, NTLNP, and VOC, respectively. These results are only slightly lower than the best‐performing Swin Transformer (0.934, 0.972, 0.988, and 0.815), yet WS‐YOLO has a significantly lower model complexity (2.69 M parameters vs. 36.8 M) and computational cost (7.1G FLOPs vs. 212.0G). Compared to other YOLO series, WS‐YOLO shows consistent improvements across all datasets, confirming the effectiveness of the proposed enhancements.

**TABLE 4:** The mAP evaluation metric results of different models on four datasets, as well as the comparison of parameter quantity and computing speed.

Model name	mAP@0.5 AW	mAP@0.5 ATRW	mAP@0.5 NTLNP	mAP@0.5 VOC	Params (M)	FLOPs (G)	FPS
Faster‐RCNN	0.905	0.914	0.927	0.712	41.35	208.1	15.8
SSD	0.857	0.872	0.912	0.688	23.75	30.4	18.9
YOLOv3‐Tiny	0.882	0.942	0.946	0.725	9.52	14.3	192.8
YOLOv5n	0.898	0.953	0.975	0.769	2.18	5.8	111.2
YOLOv6n	0.874	0.968	0.980	0.792	4.16	11.5	115.0
YOLOv8n	0.918	0.974	0.972	0.795	2.68	6.8	114.1
YOLOv9t	0.903	0.958	0.968	0.790	**1.73**	**6.4**	72.6
YOLOv10n	0.916	0.950	0.971	0.784	2.69	8.2	92.4
YOLOv11n	0.910	0.969	0.977	0.769	2.58	6.3	110.5
YOLOX‐Tiny	0.891	0.958	0.912	0.771	5.06	7.6	90.4
Swin Transformer	**0.934**	0.972	**0.988**	**0.815**	36.82	212.0	11.2
WS‐YOLO	0.930	**0.980**	0.985	0.794	2.69	7.1	121.8

*Note:* The bolded result is the best.

**TABLE 5:** The results of the *P* and *R* evaluation metric results for different models on four datasets in comparative experiments.

Model name	AW		ATRW		NTLNP		VOC	
	*P*	*R*	*P*	*R*	*P*	*R*	*P*	*R*
Faster‐RCNN	0.898	0.834	0.943	0.938	0.960	0.942	0.782	0.746
SSD	0.854	0.784	0.918	0.925	0.822	0.856	0.650	0.713
YOLOv3‐Tiny	0.863	0.810	0.923	0.933	0.959	0.934	0.740	0.656
YOLOv5n	0.878	0.826	0.958	0.949	0.964	0.945	0.772	0.675
YOLOv6n	0.910	0.833	0.944	0.946	0.957	0.953	0.779	0.718
YOLOv8n	0.911	0.848	0.931	0.948	0.952	0.948	0.784	0.705
YOLOv9t	0.892	0.820	0.951	**0.960**	0.967	0.959	0.771	0.723
YOLOv10n	0.909	0.817	0.942	0.953	0.960	0.954	0.787	0.712
YOLOv11n	0.902	0.821	0.948	0.941	0.961	0.951	0.768	0.704
YOLOX‐Tiny	0.895	0.800	0.969	0.943	0.955	0.960	0.773	0.722
Swin ransformer	0.917	0.872	0.954	0.952	**0.963**	**0.965**	**0.813**	**0.748**
WS‐YOLO	**0.922**	**0.869**	**0.972**	0.958	**0.971**	0.962	0.797	0.722

WS‐YOLO also demonstrates excellent real‐time performance with 121.8 FPS, which is among the highest across all compared models and significantly faster than Swin Transformer (11.2 FPS) and Faster R‐CNN (15.8 FPS). While YOLOv3‐Tiny shows the fastest speed (192.8 FPS), its accuracy is noticeably lower across all datasets.

Regarding precision and recall (Table 5), WS‐YOLO achieves the highest precision on 3 datasets, with values of 0.922 (AW), 0.972 (ATRW), and 0.971 (NTLNP). The recall performance is also competitive, achieving 0.869, 0.958, 0.962, and 0.722 respectively, slightly behind Swin Transformer on some datasets. Compared to YOLOv11n, both precision and recall are improved, demonstrating WS‐YOLO's enhanced ability to reduce false positives while maintaining a high true positive rate.

In summary, WS‐YOLO achieves an excellent trade‐off between accuracy, model complexity, and inference speed, outperforming most lightweight models and approaching the performance of large‐scale models like Swin Transformer, making it highly suitable for deployment in real‐world, resource‐constrained wildlife monitoring applications.

#### 3.2.2 Visual Analysis

**FIGURE 5:**
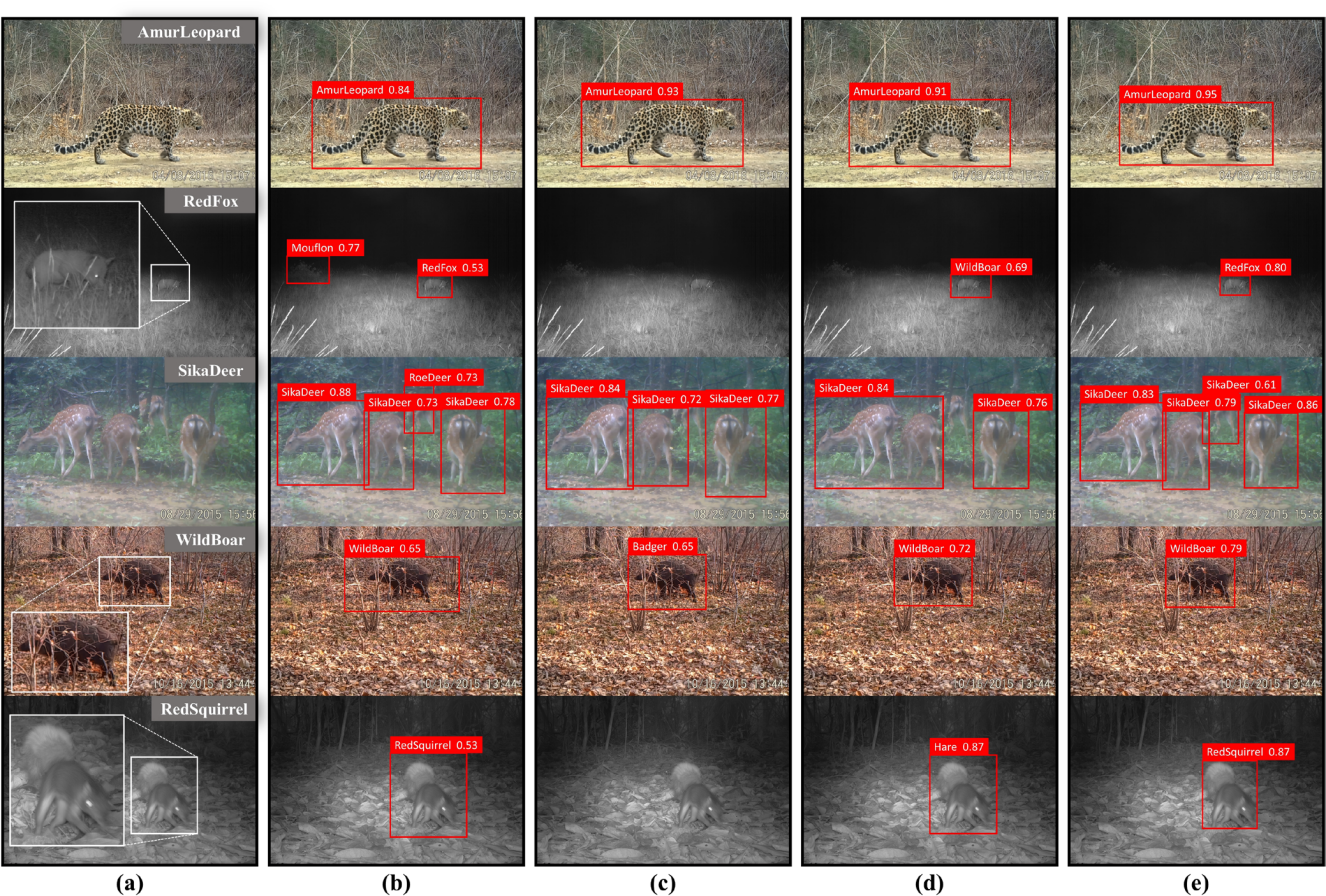
Visual comparison of detection results across different algorithms under diverse environmental conditions: (a) Original image, the species name in the upper right corner is the ground truth and the white box indicates a local magnification; (b) Faster R‐CNN; (c) Swin Transformer; (d) YOLOv11n; (e) Proposed WS‐YOLO. The five rows represent distinct scenarios: (1) large and clearly visible target; (2) small nocturnal target with background clutter; (3) multi‐object detection with occlusion; (4) partial occlusion with complex background; and (5) low‐visibility night image.

Figure 5 visually compares the detection results of five representative algorithms: Faster R—CNN, Swin Transformer, YOLOv11n, and the newly proposed WS—YOLO. Each row in this figure corresponds to a distinct detection scenario, highlighting the performance of these algorithms under different conditions related to target visibility, scene complexity, and various environmental factors.

In the first row, depicting a large and clearly visible target (an Amur Leopard), all models can successfully detect the object without issues. However, WS—YOLO stands out by yielding the highest confidence score among all models. Moreover, it generates a more precise bounding box around the Amur Leopard, demonstrating its superior certainty in target detection and exceptional localization accuracy compared to other models.

Moving on to the second row, the scenario involves a small nocturnal target, specifically a Red Fox. In this case, Faster R—CNN encounters a problem as it misidentifies background shrubs as an animal, which is a significant error. Additionally, it outputs a prediction with low confidence for the actual target, the Red Fox. Conversely, Swin Transformer fails completely in target detection. Although YOLOv11n manages to correctly identify the Red Fox, WS—YOLO not only accurately detects the target but also assigns a higher confidence score than YOLOv11n. This indicates that WS—YOLO has stronger performance capabilities, especially in low—light and low—contrast conditions where accurate detection can be quite challenging.

The third row presents a multi—object scenario involving a group of Sika Deer. Here, Faster R—CNN generates false positives, incorrectly identifying non—deer objects as deer. Meanwhile, Swin Transformer fails to detect the occluded deer within the group, which is a notable drawback. YOLOv11n performs the worst in this particular scenario as it merges adjacent objects into a single detection and misses one instance of a Sika Deer. In contrast, WS—YOLO successfully detects all individual deer with well—separated and highly accurate bounding boxes. This demonstrates its superior robustness in dense object detection, where multiple objects are in close proximity to each other.

In the fourth row, partial occlusion is introduced into the detection scenario. All models can detect the Wild Boar, but Swin Transformer misclassifies the Wild Boar as a Badger, which is a clear classification error. However, WS—YOLO provides the most accurate classification of the Wild Boar among all models. It also generates the tightest bounding box around the target and has the highest confidence score. This highlights its robustness and reliability even under conditions of partial visual obstruction, where parts of the target are not fully visible.

The fifth row focuses on noisy infrared images with low visibility. In this challenging scenario, while Faster R—CNN manages to detect the Red Squirrel, its confidence level is quite low, indicating uncertainty in its detection. Unfortunately, Swin Transformer completely misses the target, which is a major failure. YOLOv11n also performs poorly as it produces a false detection, misidentifying something as the Red Squirrel. However, WS—YOLO accurately detects the target despite the poor image quality and maintains a high confidence level in its detection. This effectively validates its effectiveness and superiority in handling such challenging low—quality inputs, where traditional models may face significant difficulties.

Overall, WS‐YOLO consistently outperforms baseline and advanced detectors across all examined scenarios, particularly in detecting small, occluded, or low‐contrast animals. These results confirm the model's enhanced capability for real‐world wildlife monitoring under complex and variable environmental conditions.

### 3.3 Edge Inference Experiment

Deploying object detection models on edge devices necessitates a careful balance between detection accuracy and computational efficiency. In particular, input image resolution plays a pivotal role in determining the trade‐off between model performance and real‐time inference capability. Although increasing input resolution generally benefits detection precision—especially in capturing fine‐grained details—it also leads to higher computational loads, which may exceed the processing capabilities of resource‐constrained devices. Conversely, reducing image size may improve inference speed but risks missing critical visual cues essential for accurate object recognition.

**FIGURE 6:**
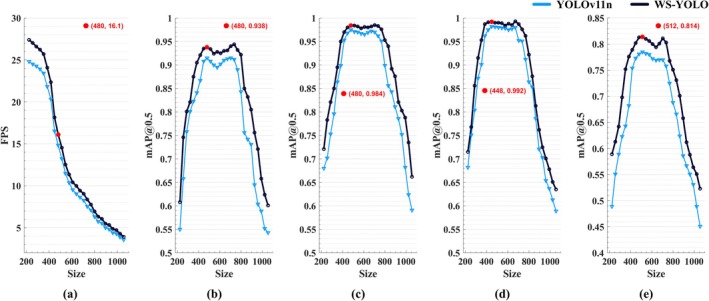
Detection mAP@0.5 results and inference speed of WS‐YOLO under varying input resolutions on NVIDIA Jetson Nano: (a) Relationship between input image resolution and inference speed (FPS) on the Jetson Nano; (b) Variation of mAP@0.5 with input resolution on the AW dataset; (c) Variation of mAP@0.5 with input resolution on the ATRW dataset; (d) Variation of mAP@0.5 with input resolution on the NTLNP dataset; (e) Variation of mAP@0.5 with input resolution on the VOC dataset.

To explore the optimal input resolution for deploying WS‐YOLO in resource‐limited environments, we conducted a series of comparative experiments. Figure 6 illustrates the experimental results of edge deployment. we evaluated the impact of different input resolutions on detection accuracy and inference speed across four datasets. The input image size was varied incrementally from 224 × 224 to 1056 × 1056, with steps of 32 pixels, and performance was evaluated in terms of detection accuracy and inference speed. Both WS‐YOLO and the baseline YOLOv11n model were tested under identical conditions.

As shown in Figure 6a, the inference speed (FPS) steadily decreases with increasing input resolution due to the higher computational demands. At a resolution of 480 × 480 pixels, WS‐YOLO achieves 16.1 FPS, representing a relatively high inference speed under edge computing constraints.

Figure 6b–e depict the mAP@0.5 curves on the AW, ATRW, NTLNP, and VOC datasets, respectively. On the AW dataset (Figure 6b), WS‐YOLO reaches a competitive mAP of 0.938 at 480 × 480, achieving a favorable trade‐off between accuracy and speed. On the ATRW dataset (Figure 6c), the highest accuracy of 0.984 is also observed at 480 × 480. The NTLNP dataset (Figure 6d) shows a peak performance of 0.992 at 448 × 448, which is very close to the results at 480 × 480. On the VOC dataset (Figure 6e), although the highest mAP of 0.814 is obtained at 512 × 512, the performance gain is marginal compared to 480 × 480, whereas the inference speed drops more sharply.

Interestingly enough, there exists a consistent and noteworthy phenomenon that can be observed across all datasets. This phenomenon is that the mAP starts to decrease at a certain point. And when does this happen? It occurs when the input resolution keeps on increasing beyond the optimal point. Now, this decline in mAP is rather counterintuitive, and there are mainly two factors that contribute to it.

Firstly, when we deal with larger input sizes, there is an introduction of interpolation noise during the resizing process. This interpolation noise is quite problematic as it has the potential to distort the features of objects within the image. And once these object features are distorted, the detection accuracy suffers a significant degradation.

Secondly, we need to consider what happens when the receptive field becomes saturated. At this stage, if we have oversized images, the additional contextual information they provide may not be as beneficial as expected. On the contrary, this extra information may bring in more background noise. And when there is an increase in background noise, the model's ability to focus on the most salient and important features is reduced. This reduction in focus on key features further contributes to the decline in mAP.

In conclusion, after taking into account both the detection performance and computational efficiency, we have determined that an input size of 480 × 480 is the most suitable resolution for WS—YOLO when it is applied on edge devices. This specific input size allows WS—YOLO to achieve robust accuracy in its detection tasks. At the same time, it also ensures that the model can maintain a relatively high inference speed, which is crucial for edge devices where computational resources may be limited and there is often a need for real—time or near—real—time processing. By selecting this optimal resolution, we can strike a good balance between the accuracy of the model and the efficiency of its operation on edge devices.

## 4 Discussion

In this study, we proposed WS‐YOLO, a lightweight detection model tailored for edge‐side wildlife image filtering and animal recognition. The model integrates WTConv, SCSA, and the adaptive SlideLoss function to address the frequent issue of false or empty triggers in infrared camera deployments. These false positives—often caused by vegetation movement, sudden illumination changes, or thermal noise—have been widely reported in camera trap deployments and remain a core challenge in remote wildlife monitoring systems (Meek et al. 2014; Ahumada et al. 2020).

WS‐YOLO builds upon the YOLOv11n baseline and introduces a frequency‐domain convolution approach to better capture fine‐grained and high frequency details, aiding in distinguishing actual animal targets from environmental noise. Previous studies have shown that multi‐scale and frequency‐aware features significantly enhance object detection robustness, particularly for small or partially occluded wildlife (Li et al. 2025). SCSA further improves model focus by jointly modeling spatial and channel‐wise attention, a strategy proven effective in ecological monitoring tasks. Although SCSA introduces additional layers, the overall model maintains low computational cost and remains suitable for real‐time inference.

The SlideLoss function is designed to adaptively adjust its focus on hard samples throughout the training process. By dynamically shifting its emphasis, it enables the model to achieve better convergence during optimization. This improved convergence not only enhances the overall training efficiency but also leads to superior detection performance, particularly in complex and challenging scenes where traditional loss functions might struggle. Such an adaptive mechanism ensures that the model can effectively handle intricate patterns and variations within the data. This behavior is consistent with findings from prior research, which have demonstrated that adaptive loss functions play a critical role in improving model generalization. Specifically, these functions are especially beneficial when dealing with longtailed or imbalanced datasets, where certain classes or samples are underrepresented. By addressing these imbalances, adaptive loss functions like SlideLoss help mitigate potential biases, thereby allowing the model to perform more robustly across diverse scenarios and ensuring fairer predictions for all classes.

The experiments conducted for the purpose of edge deployment clearly demonstrated that the WS‐YOLO model is capable of achieving an advantageous balance between accuracy and efficiency when operating at an input resolution of 480 × 480. In practical terms, this specific configuration enables the model to deliver an impressive performance of 16.1 FPSon a Jetson Nano device, which is widely recognized as a compact yet powerful platform for edge computing applications. Notably, despite the constraints of the hardware environment, the model manages to maintain high mAP scores across various datasets, underscoring its robustness and reliability in real—world scenarios.

Interestingly, further analysis revealed that increasing the input resolution beyond the 480 × 480 threshold did not yield the expected improvements in accuracy. Instead, it resulted in a noticeable decline in performance metrics. This unexpected outcome can be attributed to phenomena such as over—interpolation and noise amplification, which have been previously documented in the edge computing literature. Over—interpolation occurs when excessive data points are introduced into the system, leading to distorted predictions, while noise amplification refers to the unintended enhancement of irrelevant or erroneous information during processing. These effects collectively degrade the model's ability to make accurate predictions, thereby highlighting the importance of carefully selecting input parameters to optimize both speed and precision in edge—based deployments (Li et al. 2022).

Overall, WS‐YOLO presents a highly practical and efficient solution specifically designed for intelligent camera trap systems. By implementing this innovative approach, it effectively reduces the transmission of unnecessary images, which in turn plays a crucial role in conserving the battery life of these devices. Furthermore, WS‐YOLO significantly enhances the density of valuable data collected during long—term ecological studies. This means that researchers can obtain more meaningful and relevant information within extensive research periods, making the entire process of ecological investigation more resource—efficient and productive. Thus, the adoption of WS—YOLO in such systems brings about multiple benefits not only in terms of technical performance but also in the context of environmental research as a whole (Willi et al. 2019).

## 5 Conclusions

In this research, we introduce WS—YOLO, a lightweight and high—efficiency wildlife detection model that is specifically optimized for edge deployment within camera trap systems. Through the integration of wavelet—based convolution, spatial–channel synergistic attention, and an adaptive loss function, the model effectively eliminates false or empty images while sustaining high detection accuracy across various natural environments. Our results indicate that WS—YOLO facilitates real—time or near—real—time animal recognition on low—power devices, substantially reducing the data transmission burden and energy consumption in remote monitoring situations. This approach holds promise for improving the reliability, sustainability, and ecological utility of long—term wildlife monitoring endeavors.

## Author Contributions

**Huihui Sun:** funding acquisition (equal), methodology (equal), resources (equal), writing – original draft (equal). **Yang Liu:** data curation (equal), methodology (equal), project administration (equal), writing – original draft (equal), writing – review and editing (equal). **Yang Wang:** software (equal), visualization (equal).

## Conflicts of Interest

The authors declare no conflicts of interest.

## Data Availability

The data that support the findings of this study are openly available in African Wildlife Dataset at https://docs.ultralytics.com/datasets/detect/african‐wildlife/ (Ultralytics 2025); Amur Tiger Re‐identification in the Wild at https://www.sciencedirect.com/science/article/pii/S1470160X25001566/ (Ma et al. 2025); Northeast Tiger and Leopard National Park at https://www.mdpi.com/2076‐2615/12/15/1976/ (Tan et al. 2022).

## References

[ece373927-bib-0001] Ahumada, J. A. , E. Fegraus , T. Birch , et al. 2020. “Wildlife Insights: A Platform to Maximize the Potential of Camera Trap and Other Passive Sensor Wildlife Data for the Planet.” Environmental Conservation 47: 1–6. 10.1017/S0376892919000298.

[ece373927-bib-0002] Bochkovskiy, A. , C. Wang , and H. M. Liao . 2020. “YOLOv4: Optimal Speed and Accuracy of Object Detection.”

[ece373927-bib-0003] Bruce, T. , Z. Amir , and B. L. Allen . 2025. “Large‐Scale and Long‐Term Wildlife Research and Monitoring Using Camera Traps: A Continental Synthesis.” Biological Reviews 100: 530–555. 10.1111/brv.13152. 39822039 PMC11885691

[ece373927-bib-0004] Chung, M. A. , C. W. Lin , J. W. Lin , et al. 2025. “YOLOv3‐Based Detection Method for Sensing Railway Fastener Defect With Training Data Generated by Generative Adversarial Network Models.” Sensors and Materials 37, no. 8: 3755–3771.

[ece373927-bib-0005] Everingham, M. , L. Van Gool , C. K. I. Williams , J. Winn , and A. Zisserman . 2010. “The Pascal Visual Object Classes (VOC) Challenge.” International Journal of Computer Vision 88: 303–338. 10.1007/s11263-009-0275-4.

[ece373927-bib-0006] Finder, S. E. , R. Amoyal , E. Treister , and O. Freifeld . 2024. “Wavelet Convolutions for Large Receptive Fields.” In 18th European Conference on Computer Vision, Cham; pp. 363–380.

[ece373927-bib-0007] Jocher, G. , Q. Jing , and A. Chaurasia . 2025. “Ultralytics YOLO.” https://github.com/ultralytics/ultralytics.

[ece373927-bib-0008] Li, X. , B. Cho , and Y. Xiao . “Balancing Latency and Accuracy on Deep Video Analytics at the Edge.” In 19th Annual Consumer Communications & Networking Conference (CCNC), Las Vegas, NV, USA, 2022‐1‐1 2022; pp. 299–306.

[ece373927-bib-0009] Li, Z. , Y. Wang , and F. Wang . 2025. “DI‐YOLOv5: An Improved Dual‐Wavelet‐Based YOLOv5 for Dense Small Object Detection.” IEEE/CAA Journal of Automatica Sinica 12: 457–459. 10.1109/JAS.2024.124368.

[ece373927-bib-0010] Liu, M. , Y. Wu , R. Li , and C. Lin . 2025. “LFN‐YOLO: Precision Underwater Small Object Detection via a Lightweight Reparameterized Approach.” Frontiers in Marine Science 11: 1513740. 10.3389/fmars.2024.1513740.

[ece373927-bib-0011] Liu, W. , D. Anguelov , D. Erhan , et al. “SSD: Single Shot MultiBox Detector.” In 14th European Conference on Computer Vision, Cham, 2016; pp. 21–37.

[ece373927-bib-0012] Liu, W. , H. Liu , Y. Wang , X. Zheng , and J. Zhang . 2019. “A Novel Extraction Method for Wildlife Monitoring Images With Wireless Multimedia Sensor Networks (WMSNs).” Applied Sciences 9: 2276. 10.3390/app9112276.

[ece373927-bib-0013] Liu, Z. , Y. Lin , Y. Cao , et al. “Swin Transformer: Hierarchical Vision Transformer Using Shifted Windows.” In 2021 IEEE/CVF International Conference on Computer Vision (ICCV), 2021‐1‐1 2021; pp. 9992–10002.

[ece373927-bib-0014] Lu, X. , W. Li , and X. Lu . 2024. “An Objectness‐Aware Network for Wildlife Detection.” Multimedia Tools and Applications 83: 7119–7133. 10.1007/s11042-023-15246-8.

[ece373927-bib-0015] Ma, Y. , M. Tan , X. Liu , et al. 2025. “Deep Learning for Amur Tiger Re‐Identification in Camera Traps: A Tool Assisting Population Monitoring and Spatio‐Temporal Analysis.” Ecological Indicators 171: 113227. 10.1016/j.ecolind.2025.113227.

[ece373927-bib-0016] Meek, P. D. , G. Ballard , P. J. S. Fleming , M. Schaefer , W. Williams , and G. Falzon . 2014. “Camera Traps Can be Heard and Seen by Animals.” PLoS One 9: e110832. 10.1371/journal.pone.0110832. 25354356 PMC4212972

[ece373927-bib-0017] Megvii‐Basedetection . 2025. “YOLOX: Exceeding YOLO Series in 2021.” https://github.com/Megvii‐BaseDetection/YOLOX.

[ece373927-bib-0018] Meituan . 2025. “YOLOv6: A Single‐Stage Object Detection Framework Dedicated to Industrial Applications.” https://github.com/meituan/YOLOv6/tree/main.

[ece373927-bib-0019] Newey, S. , P. Davidson , S. Nazir , et al. 2015. “Limitations of Recreational Camera Traps for Wildlife Management and Conservation Research: A Practitioner's Perspective.” Ambio 44: 624–635. 10.1007/s13280-015-0713-1. PMC4623860 26508349

[ece373927-bib-0020] Norouzzadeh, M. S. , A. Nguyen , M. Kosmala , et al. 2018. “Automatically Identifying, Counting, and Describing Wild Animals in Camera‐Trap Images With Deep Learning.” Proceedings of the National Academy of Sciences of the United States of America 115: E5716–E5725. 10.1073/pnas.1719367115. 29871948 PMC6016780

[ece373927-bib-0021] Panigrahi, S. , P. Maski , and A. Thondiyath . 2023. “Real‐Time Biodiversity Analysis Using Deep‐Learning Algorithms on Mobile Robotic Platforms.” PeerJ Computer Science 9: e1502. 10.7717/peerj-cs.1502. PMC10495972 37705641

[ece373927-bib-0127] Pestell, A. J. L. , A. R. Rendall , R. D. Sinclair , et al. 2025. “Smart Camera Traps and Computer Vision Improve Detections of Small Fauna.” Ecosphere 16: e70220. 10.1002/ecs2.70220.

[ece373927-bib-0023] Redmon, J. , S. Divvala , R. Girshick , and A. Farhadi . 2016. “You Only Look Once: Unified, Real‐Time Object Detection.” In 2016 IEEE Conference on Computer Vision and Pattern Recognition (CVPR): Las Vegas, NV, USA, pp. 779–788.

[ece373927-bib-0024] Redmon, J. , and A. Farhadi . 2017. “YOLO9000: Better, Faster, Stronger.” In 2017 IEEE Conference on Computer Vision and Pattern Recognition (CVPR), pp. 6517–6525.

[ece373927-bib-0025] Ren, S. , K. He , R. Girshick , and J. Sun . 2017. “Faster R‐CNN: Towards Real‐Time Object Detection With Region Proposal Networks.” IEEE Transactions on Pattern Analysis and Machine Intelligence 39: 1137–1149. 10.1109/TPAMI.2016.2577031. 27295650

[ece373927-bib-0026] Si, Y. , H. Xu , X. Zhu , et al. 2025. “SCSA: Exploring the Synergistic Effects Between Spatial and Channel Attention.” Neurocomputing 634: 129866. 10.1016/j.neucom.2025.129866.

[ece373927-bib-0027] Tan, M. , W. Chao , J. Cheng , et al. 2022. “Animal Detection and Classification From Camera Trap Images Using Different Mainstream Object Detection Architectures.” Animals 12: 1976. 10.3390/ani12151976. 35953964 PMC9367452

[ece373927-bib-0028] Thu‐Mig . 2025. “YOLOv10: Real‐Time End‐to‐End Object Detection.” https://github.com/THU‐MIG/yolov10.

[ece373927-bib-0029] Ultralytics . 2025. “African Wildlife Dataset.” https://docs.ultralytics.com/datasets/detect/african‐wildlife/.

[ece373927-bib-0030] Vasavi, S. , P. N. Bandaru , and B. Sigireddy . 2025. “Edge Device Integration to Visualize Blue Whale Tracking Using Space‐Borne Remote Sensing Data.” Marine Ecology 46: e70003. 10.1111/maec.70003.

[ece373927-bib-0031] Wang, C. , A. Bochkovskiy , and H. M. Liao . 2023. “YOLOv7: Trainable Bag‐Of‐Freebies Sets New State‐Of‐The‐Art for Real‐Time Object Detectors.” 2023 IEEE/CVF Conference on Computer Vision and Pattern Recognition (CVPR), 2023‐1‐1; pp. 7464–7475.

[ece373927-bib-0032] Wang, C. , I. Yeh , and H. Mark Liao . “YOLOv9: Learning What You Want to Learn Using Programmable Gradient Information.” In 18th European Conference on Computer Vision, Cham, 2024; pp. 1–21.

[ece373927-bib-0033] Wei, W. , G. Luo , J. Ran , and J. Li . 2020. “Zilong: A Tool to Identify Empty Images in Camera‐Trap Data.” Ecological Informatics 55: 101021. 10.1016/j.ecoinf.2019.101021.

[ece373927-bib-0034] Willi, M. , R. T. Pitman , A. W. Cardoso , et al. 2019. “Identifying Animal Species in Camera Trap Images Using Deep Learning and Citizen Science.” Methods in Ecology and Evolution 10: 80–91. 10.1111/2041-210X.13099.

[ece373927-bib-0035] Wu, X. , L. Wang , and J. Huang . 2025. “AnimalRTPose: Faster Cross‐Species Real‐Time Animal Pose Estimation.” Neural Networks 190: 107685. 10.1016/j.neunet.2025.107685. 40516380

[ece373927-bib-0036] Yu, Z. , H. Huang , W. Chen , Y. Su , Y. Liu , and X. Wang . 2024. “YOLO‐FaceV2: A Scale and Occlusion Aware Face Detector.” Pattern Recognition 155: 110714. 10.1016/j.patcog.2024.110714.

[ece373927-bib-0037] Zualkernan, I. , S. Dhou , J. Judas , A. R. Sajun , B. R. Gomez , and L. A. Hussain . 2022. “An IoT System Using Deep Learning to Classify Camera Trap Images on the Edge.” Compute 11: 13. 10.3390/computers11010013.

